# Identifying mRNA targets of microRNA dysregulated in cancer: with application to clear cell Renal Cell Carcinoma

**DOI:** 10.1186/1752-0509-4-51

**Published:** 2010-04-27

**Authors:** Huiqing Liu, Angela R Brannon, Anupama R Reddy, Gabriela Alexe, Michael W Seiler, Alexandra Arreola, Jay H Oza, Ming Yao, David Juan, Louis S Liou, Shridar Ganesan, Arnold J Levine, WK Rathmell, Gyan V Bhanot

**Affiliations:** 1 BioMaPS Institute, Rutgers University, Piscataway, NJ 08854, USA; 2 Lineberger Comprehensive Cancer Center, University of North Carolina, Chapel Hill, NC 27599, USA; 3 Broad Institute of MIT and Harvard, 7 Cambridge Center, MA, 02142, USA; 4 Cancer Institute of New Jersey, Robert Wood Johnson Medical School, New Brunswick, NJ 08903, USA; 5 Department of Pathology, Boston University Medical School, Boston, MA 02118, USA; 6 Cambridge Health Alliance, Harvard Medical School, Cambridge MA 02139, USA; 7 Simons Center for Systems Biology, Institute for Advanced Study, Princeton, NJ 08540, USA; 8 Departments of Medicine and Genetics, University of North Carolina, Chapel Hill, NC 27599, USA; 9 Department of Molecular Biology and Biochemistry, Rutgers University, Piscataway, NJ 08854, USA; 10 Department of Physics, Rutgers University, Piscataway, NJ 08854, USA; 11 Current address: Bioinformatics, Centocor R&D Inc, 145 King of Prussia Road, Radnor, PA 19087, USA

## Abstract

**Background:**

MicroRNA regulate mRNA levels in a tissue specific way, either by inducing degradation of the transcript or by inhibiting translation or transcription. Putative mRNA targets of microRNA identified from seed sequence matches are available in many databases. However, such matches have a high false positive rate and cannot identify tissue specificity of regulation.

**Results:**

We describe a simple method to identify direct mRNA targets of microRNA dysregulated in cancers from expression level measurements in patient matched tumor/normal samples. The word "direct" is used here in a strict sense to: a) represent mRNA which have an exact seed sequence match to the microRNA in their 3'UTR, b) the seed sequence match is strictly conserved across mouse, human, rat and dog genomes, c) the mRNA and microRNA expression levels can distinguish tumor from normal with high significance and d) the microRNA/mRNA expression levels are strongly and significantly anti-correlated in tumor and/or normal samples. We apply and validate the method using clear cell Renal Cell Carcinoma (ccRCC) and matched normal kidney samples, limiting our analysis to mRNA targets which undergo degradation of the mRNA transcript because of a perfect seed sequence match. Dysregulated microRNA and mRNA are first identified by comparing their expression levels in tumor vs normal samples. Putative dysregulated microRNA/mRNA pairs are identified from these using seed sequence matches, requiring that the seed sequence be conserved in human/dog/rat/mouse genomes. These are further pruned by requiring a strong anti-correlation signature in tumor and/or normal samples. The method revealed many new regulations in ccRCC. For instance, loss of miR-149, miR-200c and mir-141 causes gain of function of oncogenes (KCNMA1, LOX), VEGFA and SEMA6A respectively and increased levels of miR-142-3p, miR-185, mir-34a, miR-224, miR-21 cause loss of function of tumor suppressors LRRC2, PTPN13, SFRP1, ERBB4, and (SLC12A1, TCF21) respectively. We also found strong anti-correlation between VEGFA and the miR-200 family of microRNA: miR-200a*, 200b, 200c and miR-141. Several identified microRNA/mRNA pairs were validated on an independent set of matched ccRCC/normal samples. The regulation of SEMA6A by miR-141 was verified by a transfection assay.

**Conclusions:**

We describe a simple and reliable method to identify direct gene targets of microRNA in any cancer. The constraints we impose (strong dysregulation signature for microRNA and mRNA levels between tumor/normal samples, evolutionary conservation of seed sequence and strong anti-correlation of expression levels) remove spurious matches and identify a subset of robust, tissue specific, functional mRNA targets of dysregulated microRNA.

## Background

MicroRNA regulate mRNA and protein levels by cleavage and/or translation/transcriptional repression in a tissue specific manner [1-4]. By modulating key cellular processes such as metabolism, division, differentiation, development and apoptosis, they can simultaneously regulate both oncogenes and tumor suppressor genes [5-7]. Aberrant microRNA profiles have been noted in many cancers [5-11], including renal cell carcinoma [12-16]. Almost half the known microRNAs are in cancer-associated chromosomal fragile sites, susceptible to point mutation, amplification, deletion, or translocation [17,18]. Recent evidence demonstrates that microRNA play an important role in the patho-physiology of many cancers [19-22] and they are believed to be involved in pathogenesis in ccRCC [20,23]. MicroRNA are also being studied in various tumors to understand their significance for drug resistance [24,25], diagnosis and prognosis [26-28] and for their therapeutic potential [29-40]. Their secondary structure preserves them better in FFPE samples than mRNA, making them easier to extract in intact form, resulting in higher identification accuracy in the analysis of archived clinical material [35]. Their tissue specificity and tight regulation makes them more reliable identifiers of tissue of origin in highly differentiated tumors [41]. Single microRNA can regulate multiple mRNA and are therefore both better identifiers of mechanism and possibly better drug targets [4]. However, while it is clear that microRNA play an important role in the biology of many cancers, their complex biology and tissue specificity makes it difficult to understand the precise role they play in the disease process and the genes affected by their dysregulation [42-47].

Mature microRNAs are produced in a multi-stage process. After transcription, they are processed by RNA Pol II or Pol III to create capped and polyadenylated primary transcripts (Pri-microRNAs), which are further processed in the nucleus by the enzyme Drosha/Pasha (in flies) or by DGCR8 (in humans) to produce ~60-nucleotide Pre-microRNA stem-loop molecules. These are then exported to the cytoplasm by Exportin and Ran-GTP where they are further processed by Dicer to ~22 nt double-stranded RNA duplexes, which form complexes with RISC (RNA-induced silencing complex) leading to unwinding of the duplexes to form single-stranded microRNAs. MicroRNAs bound to RISC can down-regulate protein levels using at least two alternative pathways: 1) If the microRNA has imperfect complementarity with a matching sequence in the 3'UTR of its target mRNA, the microRNA-RISC complex can combine with the complement mRNA sequence and cause translational repression. 2) On the other hand, if the microRNA and its mRNA target have perfect or near perfect complementarity, the microRNA-RISC complex binding to its target mRNAs can result in the cleavage and degradation of the mRNA by Argonaute2 (Ago2) [1-7,35].

Although many studies have identified signatures of microRNA dysregulation, the identification of tissue specific targets of aberrantly regulated microRNA is difficult. Putative identification using seed sequence complementarity and free energy predictions of RNA-RNA duplexes [48-55] are available in databases such as TargetScan: http://www.targetscan.org. However, the false positive rate for such matches is unacceptably high, with different algorithms identifying different mRNA targets for the same microRNA [51-53,56,57]. The tissue specificity of microRNA regulation is known only in some specific cases (e.g. see Table one in [58]) and a general methodology for target identification, tissue specificity of action and specific biological role of microRNA in the initiation and progression of most cancers remains an open problem.

We describe a novel method to identify "direct mRNA targets" of microRNA in any cancer based on measuring an anti-correlation signal between differentially expressed microRNA and mRNA in patient matched tumor and normal samples. In this paper, the words "direct mRNA targets" is used in a very strict and limited sense. A direct target is one which: a) has an exact seed sequence match in its 3'UTR to the corresponding microRNA, b) the seed sequence match is conserved across mouse, human, rat and dog genomes, c) the expression levels of both the microRNA and the mRNA can distinguish tumor from normal with high statistical significance and d) the mRNA and microRNA levels are strongly and significantly anti-correlated in tumor and/or normal. These requirements could be relaxed to find additional targets or eliminated altogether to find indirect regulations (see later discussion).

The method proceeds as follows: a) Identify significantly differentially expressed microRNA and mRNA between the two classes (e.g. normal and tumor); b) For each microRNA which is differentially expressed, identify all its putative target mRNA by restricting to those differentially expressed mRNA with a matching seed sequence in their 3'UTR, with the further requirement that it be conserved in human, mouse, rat and dog genomes; c) Compute the Pearson correlation between microRNA and mRNA expression levels for samples in each class (tumor and normal) and d) Retain only those microRNA/mRNA pairs whose expression levels are highly anti-correlated. These constraints remove spurious matches, reducing relatively speculative "putative" seed match based mRNA targets in databases to a highly robust subset of direct functional targets.

Note that our method can be extended (with data on more samples) by removing constraint b) and looking for a correlation (or anti-correlation) signature in c). This allows the identification of indirect regulation. For example, if a microRNA up-regulated in cancer down-regulates a gene which is a transcriptional repressor of an oncogene, then the expression level of the microRNA will be correlated with the level of the oncogene without a seed sequence match. Since the direct gene target (the transcriptional repressor in the example above) of the microRNA should already be identified using our method, such an analysis would extend the regulation network beyond first order interactions. Note that, although the method as described above does not identify regulation by translation inhibition (because this would not significantly affect mRNA levels), if protein levels were also measured, the method could easily be extended to identify such regulation.

We demonstrate the use of our method on expression data from clear cell Renal Cell Carcinoma (ccRCC) and matched normal kidney samples. Renal Cell Carcinoma (RCC) represents ~3% of all malignancies in the US, with 50,000 new cases and 12,000 deaths each year http://www.nci.nih.gov/cancertopics/types/kidney. The most common histological class is ccRCC, accounting for ~75% of kidney cancers. ccRCC is known to be characterized by the loss of the VHL gene, which under normal oxygen pressure, binds to the α subunits of hypoxia-inducible factors (HIFs), inducing their poly-ubiquitinylation and subsequent degradation in the proteasome. In hypoxic conditions, or if HIF regulation is lost because of VHL inactivation, HIF accumulates to high levels and promotes the transcription of genes such as VEGF, PDGF-β, TGF-α, EPO etc which trigger angiogenesis, cell growth, migration and proliferation [59,60]. The spectrum of HIF target genes expressed in individual tumors and the factors which influence them are the object of active ongoing research. ccRCC tumors have a wide range of natural histories and varied responses to VEGF-targeted therapy [61]. Early stage, Fuhrman grade 1 (low grade) tumors tend to have significantly better disease free survival after resection than higher stage and grade (Fuhrman grade 4) [62]. Although VHL mutation is associated with all grades of ccRCC, the other molecular factors associated with ccRCC initiation and progression are largely unknown. The molecular basis of the diversity in histologic grade, clinical behavior, and response to VEGF-targeted is also unclear, and makes ccRCC a ripe target for studies investigating the molecular and genetic nature of these heterogeneities.

In RCC, various studies have identified panels of microRNA and mRNA that are differentially expressed between normal renal tissue and tumor or between histological subtypes of tumor [12,14,15,63-66]. The present study extends these previous studies by linking the microRNA to some of their mRNA targets, thus elucidating a hitherto unknown part of the biology of ccRCC disease. Some of the identified microRNA/mRNA anti-correlations were validated on a new cohort of ccRCC/normal samples. SEMA6A was confirmed as a direct target of miR-141 by over-expressing miR-141 in a ccRCC cell line and showing strong down-regulation of the SEMA6A transcript.

## Results

The underlying hypothesis in our method is that the expression levels of microRNA and their *direct *mRNA targets should be *strongly anti-correlated *when averaged over matched samples in either tumor or normal tissue. The stepwise procedure is as follows:

Step 1: Identify significantly up/down regulated microRNAs in ccRCC samples vs normal samples.

Step 2: Identify significantly up/down regulated mRNAs in ccRCC samples vs normal samples.

Step 3: Using TargetScan, retain only the mRNA in Step 2 which have a *conserved *seed sequence in their 3' UTR for at least one of the microRNA from Step 1.

Step 4: Find anti-correlated pairs of up-regulated microRNA and down-regulated mRNA in ccRCC samples using a strict cutoff (P_0_) in Pearson correlation coefficient in ccRCC samples. Similarly, find anti-correlated pairs of down-regulated microRNA and up-regulated mRNAs in normal kidney samples using a strict cutoff.

In Step 3, putative target mRNA were identified using TargetScan Version 4.1 http://www.targetscan.org, which identifies possible regulatory targets of mammalian microRNAs as those with conserved sequences of matching seed regions for each microRNA. The term "*conserved*" means that the sequence is conserved in human, mouse, rat and dog. In Step 4, we ran 1000 permutations in BRB-ArrayTools' http://linus.nci.nih.gov/BRB-ArrayTools.html and multivariate/univariate analysis at *p *< 0.01, FDR < 0.2 to assess significance of discovered pairs and to find the appropriate cutoff P_0 _for significance of the measured Pearson correlation. In the primary dataset, because of the high accuracy of qRT-PCR, we were able to set a strict cutoff P_0 _= -0.95; thus in Step 4 only microRNA/mRNA pairs with P < P_0 _(= -0.95) were considered to be significant.

### Step 1: MicroRNA significantly differentially expressed in ccRCC versus normal kidney tissue

**Figure 1:**
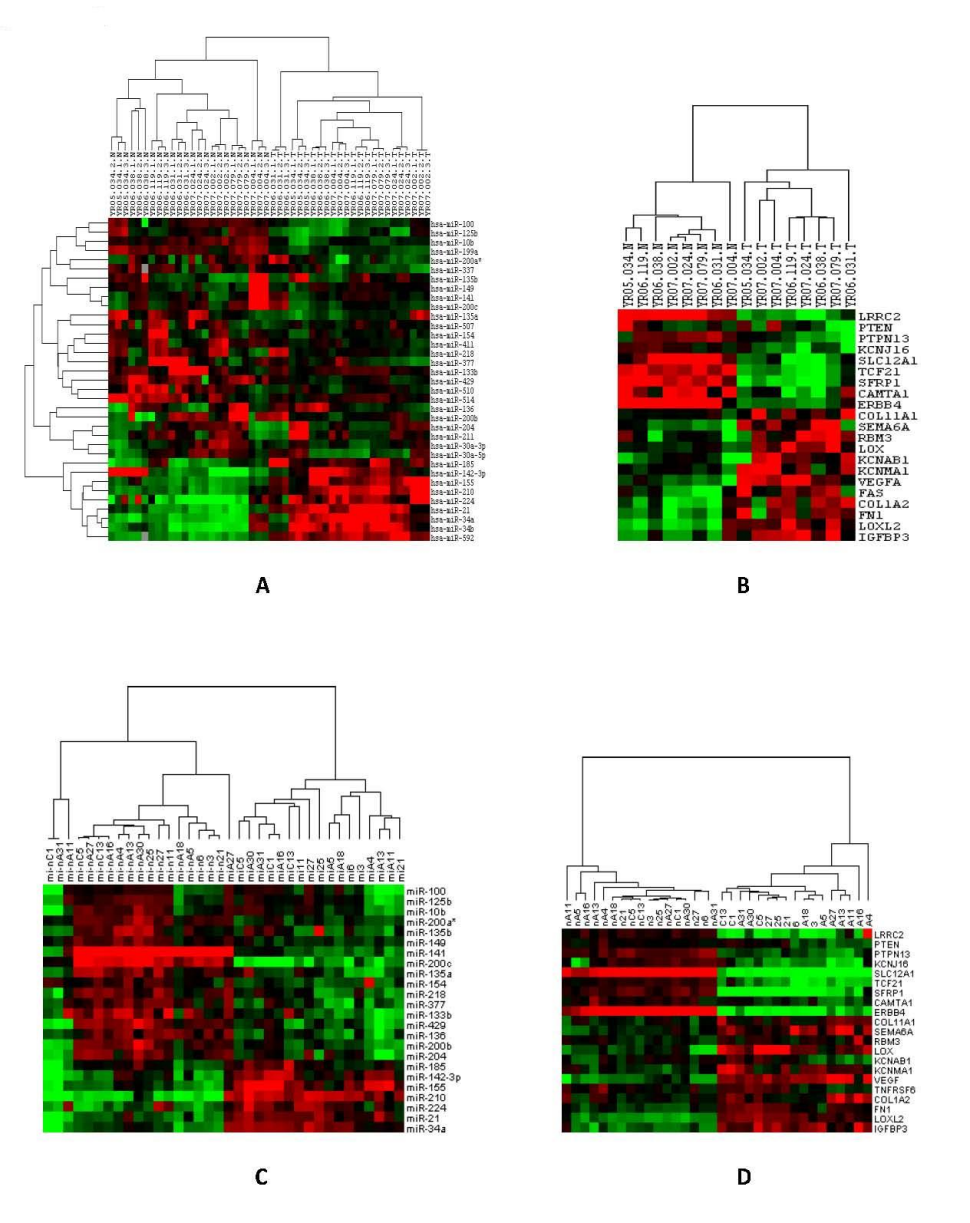
**Differential expression of microRNA and mRNA between normal and tumor tissue**. A. Heatmap of 35 microRNA differentially expressed in ccRCC and matched normal kidney tissue in the primary dataset. B. Heatmap of some of the identified mRNA targets of the microRNA in A as identified by our method. C. Heatmap of the 27 microRNA in A which were available on the Agilent chipset used on the validation samples. D. Validation sample set heatmap of mRNA levels for the same genes as in B.

35 microRNA were identified as differentially expressed (p < 0.001) in ccRCC versus normal kidney, 26 down-regulated and 9 up-regulated. The microRNA down-regulated in ccRCC were miR-100, miR-10b, miR-125b, miR-26a+, miR-133b, miR-135a, miR-135b, miR-136, miR-141, miR-149, miR-154, miR-199a, miR-200a, miR-200b, miR-200c, miR-204, miR-211, miR-218, miR-30a-3p, miR-30a-5p, miR-337, miR-411, miR-429, miR-507, miR-510, miR-514 and the microRNA up-regulated in ccRCC were miR-142-3p, miR-155, miR-185, miR-21, miR-210, miR-224, miR-34a, miR-34b, miR-592 (see Table 1), in agreement with recent studies [12,14,15,65,66]. The chromosomal location of these microRNA (Table 1) shows that several are proximal to fragile regions and regions commonly deleted in ccRCC [17,18]. Figure 1A shows a heat-map of the microRNA expression levels for samples in the primary dataset showing that they can robustly separate ccRCC from normal kidney.

**Table 1:** The 35 microRNA that distinguish tumor from normal tissue in human ccRCC.

microRNA	Expression Status in ccRCC	Hystotype (from references: [5-22,35-40,65,66,73,74,79])	**Cancer-related Regions **[17,18]
			
Name			
miR-100	Down	Up in pancreas, stomach	11q23-q24 (D)
			
		Down in ovarian	

miR-10b	Down	Down in breast	

miR-125b	Down	Up in pancreas	11q23 (D)
			
		Down in breast	

miR-26a^+^	Down	Down in epithelial cancers	3p21.3 (D)

miR-133b	Down	Down in ovarian	

miR-135a	Down		3p21.1-21.2 (D)

miR-135b	Down		

miR-136	Down		14q32 (D)

miR-141	Down	Up in lung, ovarian	

miR-149	Down		2q37 (D)

miR-154	Down	Down in ovarian	14q32 (D)

miR-199a	Down	Up in lung, pancreas, prostate	
			
		Down in ovarian	

miR-200a*	Down	Up in ovarian	

miR-200b	Down	Up in lung, ovarian	

miR-200c	Down	Up in ovarian	

miR-204	Down	Down in ovarian	

miR-211	Down		

miR-218	Down		4p15.3 (D)

miR-30a-3p	Down		

miR-30a-5p	Down	Down in lung	

miR-337	Down		14q32 (D)

miR-377	Down		

miR-411	Down		14q32 (D)

miR-429	Down		

miR-507	Down		

miR-510	Down		

miR-514	Down		

miR-142-3p	Up		

miR-155	Up	Up in breast, colon, lung	21q21 (A)

miR-185	Up	Up in kidney, bladder	

miR-21	Up	Up in breast, colon, lung, pancreas, prostate, stomach,; gliobastoma cervical	17q23.2 (A)

miR-210	Up	Up in breast	

miR-224	Up	Down in lung, ovarian	Xq28

miR-34a	Up	Up in lung, rat RCC	11q23-q24 (D)
			
		Down in neuroblastoma	

miR-34b	Up		

miR-592	Up		

Cancer-related regions listed are either deleted regions (D), amplified regions (A) or breakpoint regions.

### Step 2: mRNA differentially expressed in ccRCC versus normal kidney tissue

Using SAM http://www-stat.stanford.edu/~tibs/SAM/ at FDR = 0.1 and 100 permutation tests, 2632 mRNA probes were found up-regulated and 2238 were found down-regulated in ccRCC. The genes represented by these probes included many genes known to be dysregulated in ccRCC [63,64]. We saw significant upregulation of oncogenes VEGFA (vascular endothelial growth factor) and EGFR (epithelial growth factor receptor), integrin-mediated cell adhesion pathway genes (ITGA3, IGA5, ITGAM, ITGAX, ITGAL and CAV1), cell adhesion related genes (FN1, COL4A and LAMA4) and cytokines (GBP2 and GBP5). Similarly we noted down-regulation of tumor suppressor genes (VHL, SFRP1, CDKN1C and S100A2) and of members of the metallothionein family (MT2A, MT1E, MT1F, MT1G, MT1H, MT1M, MT1X).

### Steps 3 and 4: Identifying direct mRNA targets of dysregulated microRNA

Pearson correlation analysis with P_0 _= -0.95 was applied to each of the 35 differentially expressed microRNA and its putative mRNA targets (those with conserved seed sequences in their 3'UTR as found in TargetScan). This procedure identified 11 mRNA targets for the 9 up-regulated microRNA. This list included several important tumor suppressor genes, such as PTEN, ERBB4 and SFRP1, known to be mutated or down-regulated in many tumors, including ccRCC [16,17,23]. The 26 down-regulated microRNA had 170 direct up-regulated mRNA targets, including oncogenes VEGFA, LOX, LOXL2 and FAS, well known to be involved in kidney cancer [16,17,23].

**Figure 2:**
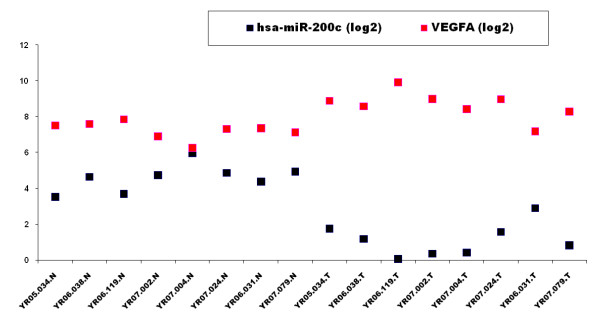
**Anti-correlation of microRNA expression and predicted target mRNA**. Expression levels of miR-200c and VEGFA in the primary dataset showing anti-correlation in both ccRCC and matched normal kidney tissue. This plot suggests that loss of miR-200c function in ccRCC contributes to increase in VEGF levels.

The nine most significantly down-regulated and twelve most significantly up-regulated mRNA are listed in Table 2 and their heatmap is shown in Figure 1B. In Figure 2 we plot microRNA and mRNA levels for miR-200c and its target VEGFA. Note that the levels of miR-200c and its target VEGFA are not only anti-correlated overall, but are also anti-correlated separately in both ccRCC and normal tissue. Additional Files 1 and 2 contain the full list of microRNA/mRNA regulations identified by our analysis.

**Table 2:** Some representative, direct mRNA targets of microRNA predicted by our method.

microRNA	mRNA	Status of mRNA in ccRCC
miR-142-3p	LRRC2	Down ^+^

miR-185	PTEN	Down

miR-185	PTPN13	Down ^+^

miR-185	KCNJ16	Down

miR-21	SLC12A1	Down ^+^

miR-21	TCF21	Down ^+^

miR-34a	SFRP1	Down ^+^

miR-34a	CAMTA1	Down

miR-224	ERBB4	Down ^+^

miR-199a	COL11A1	Up

miR-141/200a*	SEMA6A	Up ^+^

miR-141/200a*	RBM3	Up

miR-149	LOX	Up ^+^

miR-149	KCNAB1	Up ^+^

miR-149	KCNMA1	Up ^+^

miR-200bc/429	VEGF	Up ^+^

miR-200bc/429	FAS	Up

miR-204/211	COL1A2	Up

miR-204/211	FN1	Up

miR-218	LOXL2	Up

miR-218	IGFBP3	Up

The full list of identified microRNA/mRNA pairs is given in Additional Files 1 and 2. ^+ ^marks microRNA/mRNA anti-correlations that were tested/validated on an independent set of matched ccRCC/normal kidney samples.

### Validation on a cohort of ccRCC/normal kidney samples

#### A. Validation of differentially expressed microRNA and mRNA

Seventeen validation samples were collected from a new cohort of patients and analyzed by microRNA and mRNA profiling on Agilent arrays (see the Methods Section). Figure 1C shows a heat map of twenty-seven microRNA (those which were found on the Agilent chip) of the thirty five differentially expressed microRNA identified previously. A weighted voting classifier on binarized microRNA expression data in the validation set had 100% accuracy in leave-one-out (LOO) cross validation in distinguishing ccRCC from normal kidney. Figure 1D shows a heatmap of mRNA expression levels for the genes in Figure 1B. These were also found to be 100% accurate at discriminating ccRCC from normal kidney using weighted voting and LOO cross validation analysis.

**Figure 3:**
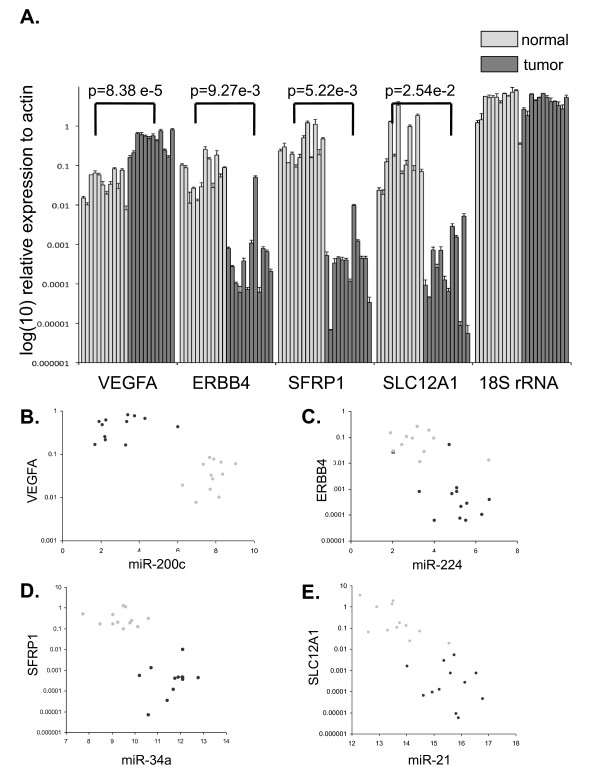
**qRT-PCR validation of predicted mRNA targets of microRNA**. **A**. qRT-PCR measured mRNA levels of VEGF, ERBB4, SFRP1, and SLC12A1 in 12 ccRCC and matching normal kidney tissue in the validation set. Expression levels, including those of 18S rRNA (control) are plotted on a log-scale relative to a housekeeping gene (beta-actin). These expression level changes agree with predicted changes based on the primary dataset. Dark grey bars denote tumors, while light grey bars denote normal kidney tissue. p-values are for accuracy of discrimination of ccRCC/normal kidney using the t-test. **B-E**. Agilent chip expression levels of miR-200c, miR-244, miR-34a and miR-21 versus the levels of mRNA that they regulate: VEGFA, ERBB4, SFRP1 and SLC12A1 respectively as measured by qRT-PCR for 12 validation set samples The dark circles represent the values in ccRCC and the light circles in normal kidney. It is clear that loss of mir-200c regulation contributes to an increase in VEGFA transcript while for the other three (tumor suppressor genes), the level of transcript decreases because of a gain in the level of the corresponding microRNA.

mRNA levels of three genes (ERBB4, SFRP1, SLC12A1) which were down-regulated in ccRCC and one gene (VEGFA) up-regulated in ccRCC were also measured by quantitative RT-PCR in twelve of the test samples. The results, shown in Figure 3A, demonstrate that mRNA levels of ERBB4, SFRP1, SLC12A1 and VEGFA were quantitatively and significantly down/up-regulated as expected.

#### B. Validation of anti-correlation signature between some identified microRNA/mRNA pairs

**Figure 4:**
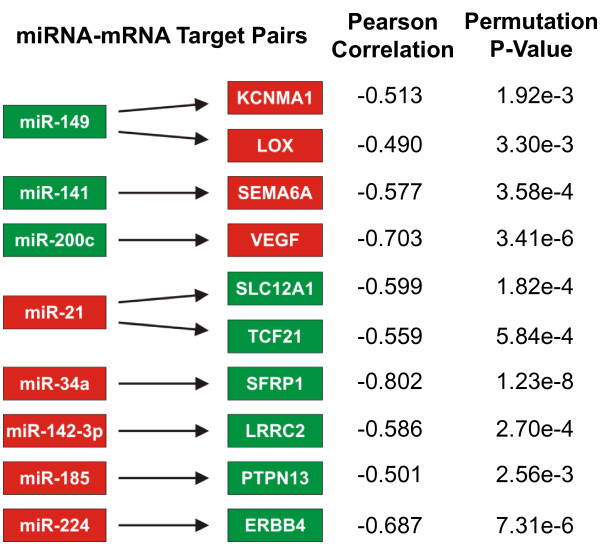
**Validation of microRNA/mRNA regulation relationships in Table 2**. Red/green boxes represent over/under expression of microRNA or mRNA levels in ccRCC compared to normal kidney. Pearson correlations were calculated for mRNA and microRNA expression values for tumor and normal combined and are shown with their p-values for significance using the permutation test.

In Figure 3B-E, we plot the qRT-PCR expression levels of ERBB4, SFRP1, SLC12A1 and VEGFA versus Agilent chip measured levels of their regulatory microRNA (miR-224, miR-34a, miR-21 and miR-200c) for the twelve samples of Figure 3A. The overall strong anti-correlation signature between microRNA and mRNA levels is clearly visible in these plots. Figure 4 summarizes our validation analysis of a number of anti-correlation measurements between several identified microRNA/mRNA pairs in the Agilent chip data. The measured correlations between predicted microRNA/mRNA pairs are shown in the figure. Because of the higher level of noise in the Agilent chip data compared to qRT-PCR, we cannot apply the strict criterion (P_0 _= -0.95) used in the primary dataset. Instead, the significance of the correlation (also shown) was computed using permutation tests as follows: A large number of datasets were obtained by permuting the sample labels in the microRNA or mRNA measurements. For each microRNA/mRNA pair, these permuted datasets were used to compute the null distribution for P and the significance of the measured value of P was estimated in this null distribution. As the p-values in Figure 4 indicate, we validate a strong anti-correlation signature between mRNA levels of (KCNMA1, LOX), VEGF, SEMA6A, (LRRC2, PTPN13), SFRP1, ERBB4, SLC12A1 and TCF21, and their identified regulators: miR-149, miR-200c, mir-141, miR-142-3p, miR-185, mir-34a, miR-224 and miR-21 respectively.

#### C. In vitro validation of SEMA6A as a target of miR-141 in an RCC cell line

**Figure 5:**
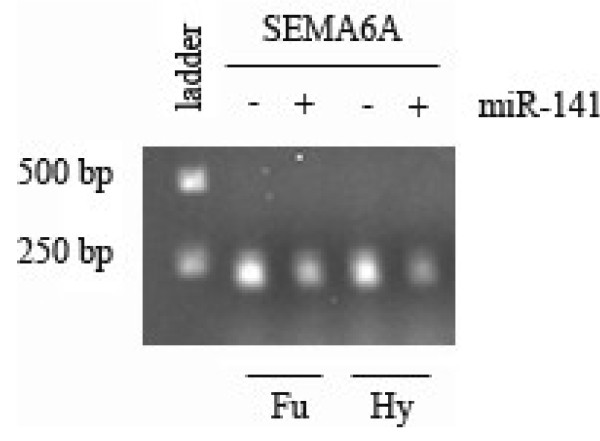
**Validation of a Direct interaction between miR-141 and SEMA6A**. Results of in-vitro experiment on RCC cell line CRL-1611 transfected either with pre-miR-141 or a control pre-miR using either Fugene (Fu) or Hyfect (Hy). RNA was extracted after 48 hours and the expression of SEMA6A was measured using semi-quantitative RT-PCR. There is clear reduction in mRNA of SEMA6A upon introduction of miR-141 by either transfection method in these cells.

Finally, to establish that this method can accurately predict functional and direct microRNA/mRNA regulation, we performed an in vitro analysis of one microRNA (miR-141), and its identified direct target SEMA6A. The RCC cell line CRL-1611 was transfected (by either Fugene or HyFect methods) with either pre-miR-141 or a control pre-miR, and levels of SEMA6A were measured on the case/control cell lines by semi-quantitative RT-PCR. The results (Figure 5) showed that introduction of pre-miR-141 produced a significant reduction in the level of SEMA6A mRNA, validating SEMA6A as a functional and direct target of miR-141.

## Discussion

We have developed and validated a simple method to identify direct functional mRNA targets of microRNA in ccRCC using patient matched tumor/normal samples. The method looks for the subset of anti-correlated microRNA/mRNA pairs from a larger set of microRNA and mRNA able to distinguish tumor from normal tissue, with the additional requirement of a highly conserved seed sequences for the corresponding microRNA in the 3'UTR of the corresponding gene. Our method can not only identify microRNA/mRNA pairs which *discriminate *normal from cancer tissue, but also dysregulated regulation mechanisms between them that may initiate and/or drive the disease process.

We used an RT-PCR panel for our initial discovery experiments and microRNA and microarray chips both for validation of our previously discovered microRNA/mRNA pairings and to expand the available pool of microRNAs to all currently known microRNAs for a comprehensive examination of microRNA/mRNA pairs. In comparing the two data modalities, we noticed that in the RT-PCR data, the sample to sample variation in the normal samples (i.e. the population variation) was comparable to the change in level between normal and tumor (in the same patient) which caused a statistically significant up/down regulation of the target mRNA. To find reliable matches, it was therefore crucial to minimize stochastic error. We observed that the RT-PCR data had lower stochastic variance than the microarray data (estimated using replicate measurements in the RT-PCR analysis and from bootstrap analysis of the microarray data). This noise effect is also reflected in the measured P0 values in the validation analysis on the microarray data (Figure 4), which are smaller in magnitude (|P0|~0.5-0.6) than the strict cutoff |P0| > 0.95 used in the discovery phase on the RT-PCR data. Since the present study is mainly to demonstrate "proof of concept", we limited the RT-PCR data for discovery and the microarray data for validation. In a more extensive study, with sufficiently large sample sizes and more accurate data from high throughput technologies (microarrays, sequencing) it may be feasible and cost effective to use a high throughput platform for discovery and RT-PCR for validation. Such an analysis might also identify a much bigger set of microRNA/mRNA relationships. Although our method is very robust, as described, it cannot find all mRNA targets. Its principal merit is the ability to reduce the large number of relatively speculative matches from seed sequences alone to a smaller set of functional, tissue specific targets. While this smaller set is perhaps incomplete, it is more reliable. Additionally, the use of perfect seed sequence matches can easily be relaxed. A more serious issue is that the method as described cannot find targets affected by translation inhibition. For such targets, the changes in microRNA levels would leave mRNA levels unaffected, but reduce protein levels. Such targets might be identified if protein levels in tumor/normal samples were also measured along with the microRNA and mRNA levels. The method could then be adapted to identify targets affected by translation inhibition by using an appropriate joint correlation/anti-correlation analysis of microRNA/mRNA/protein levels. Our method, as described here, would also miss regulation that proceeds via intermediate states. In these cases, there would be no seed sequence match (exact or approximate) between a microRNA and the mRNA whose level was affected by it. We could attempt to identify such secondary effects by eliminating the seed sequence match requirement but keeping the requirement of a high correlation/anti-correlation signal between microRNA/mRNA levels. Whether such a procedure would work would depend on the degree to which the intermediate state gene/protein is affected and measurable. We briefly discuss some of these issues below. However, it should be noted that these studies would require significantly larger sample sizes and are beyond the scope of the present paper. The next round of TCGA http://cancergenome.nih.gov/ may make these types of analyses more feasible.

Although here, we focused on microRNA/mRNA pairs with exact seed sequence match and an anti-correlation signal in both tumor and normal samples, as noted above, with additional samples it should be possible to use this method and simple extensions to identify more subtle types of dysregulation. For example, if a mutation in a microRNA in the tumor samples causes loss of its function (failure to regulate its target mRNA), then although the microRNA/mRNA levels would be anti-correlated in normal tissue, they would not be anti-correlated in tumor samples. Similarly a de-novo gain of microRNA function would be signaled by an anti-correlated signal in tumor samples which was absent in the normal samples.

In our data, we did observe several correlations and anti-correlations between microRNA/mRNA pairs in tumor or normal samples without a corresponding seed sequence match. As noted above, these most likely represent regulations which proceed via intermediate states and hence cannot be identified by seed sequence matches. For example, if increased levels of microRNA X down-regulates expression of a protein which is a transcriptional repressor of gene Y, there will be a strong correlation between X and Y levels but no seed sequence match. Conversely, if microRNA X regulates a protein which is a transcription factor for gene Y then the levels of X and Y will be anti-correlated without a seed sequence match. The measurement of such correlations would extend the network of microRNA control beyond first level regulators but would require significant increases in the number of samples (~100-200) for statistical significance.

Since the method finds functional relationships, it should be useful for identifying pharmaceutically relevant mechanisms which suggest drug targets for therapy. We describe below some of the regulations we identified which have pharmaceutical relevance.

### microRNA which shut down multiple tumor suppressor genes in ccRCC

We found several dysregulated microRNA which targeted multiple tumor suppressor genes. For example, the oncogenic miR-185 was significantly up-regulated in ccRCC and anti-correlated with the tumor suppressor gene PTEN, suggesting that its gain of function shuts down PTEN in ccRCC. Mutated or down-regulated in many advanced cancers [67], PTEN loss activates the PI3K-AKT [68] signaling pathway and its downstream target mTOR, with important implications in RCC development and therapeutic selection [59,60,69,70]. Another identified target of miR-185 was PTPN13 (also a predicted miR-185 target in miRBase: http://microrna.sanger.ac.uk/), which is a Fas-associated protein tyrosine phosphatase and putative tumor suppressor gene that can inhibit PI3K/AKT signaling, suppress the influence of insulin-like growth factor-I on cell survival and induce apoptosis [71]. KCNJ16, member of the potassium channel subfamily of membrane proteins, was also identified as a target of miR-185. Such membrane proteins have been suggested in anti-cancer therapies because of their important role in cell growth [72] and are known to be down-regulated in RCC [73].

miR-34a is known to be over-expressed in various tumors and associated with cell proliferation [74]. In our data, it was up-regulated in ccRCC and predicted to target SFRP1, a known regulator of the Wnt signaling pathway and a tumor suppressor gene whose loss has been observed in a majority of RCC patients [75]. Another tumor suppressor regulated by miR-34a was CAMTA1 [76], a reduction in whose levels correlates with poor outcome in neuroblastoma [77]. Finally, we find miR-224, associated with a human chromosome fragile site on Chr-Xq28 [18], is up-regulated in ccRCC and predicted to target ERBB4, a member of the EGFR family, a potential tumor suppressor known to be strongly down-regulated in ccRCC [78].

### Hypoxia induced microRNAs

A hypoxic tumor microenvironment can directly activate the expression of several microRNA [79,80]. For example, miR-21, miR-210 and miR-155 reduce pro-apoptotic signaling in response to a hypoxic environment and are consistently over-expressed in a variety of human tumors (Table 1). ccRCC is a unique setting in which to study these microRNAs, given that *VHL *loss constitutively stabilizes one or more HIF factors, thereby creating a pseudo-hypoxic scenario in ccRCC tumor cells [59,60]. We found that hypoxia related microRNA had the most significant fold changes, with miR-210, miR-155 and miR-21 being amongst the top., suggesting a major role for them in renal carcinogenesis. We found 16 genes down-regulated in tumor, inversely correlated with miR-21 and enriched (via KEGG) in the cell adhesion (CAM) pathway at *p *= 0.0006. Loss of CAM degrades the intra and extracellular matrix, leading to abnormal cell growth patterns. Among these 16 genes, two were identified as direct targets of miR-21: SLC12A1 and TCF21, both of which have been reported down-regulated in ccRCC [69,70].

### Identification of microRNA family interactions

We found evidence that families of microRNAs may be coordinately participating in microRNA/mRNA interactions. One example in ccRCC is the miR-204/211 family, which was significantly down-regulated in ccRCC samples. We identified thirty five significant mRNA targets (*p *= 0.0001) for this family (Additional File 2). Among these, eight were on Chromosome 3q, a common amplicon region in many epithelial tumors [81]. These genes include C3orf58, CCDC50, DTX3L, PLD1, TRIM59, two oncogenes ECT2 and RAP2B, and a hypoxia associated protein SERP1 [82]. The gain of this chromosome arm was previously associated with papillary RCCs [83], and our observation in clear cell RCC implies a possible regulatory relation between miR-204/211 and the genes in this region as an alternate mechanism of up-regulation of this group of genes.

Another example is the miR-200 family which includes two microRNA clusters, one on Chromosome 1p36.3 (miR-200a*/200b/429) and another on Chromosome 12p13 (miR-200c/141). Five miR-200 family members contain very similar seed sequences - AAUACU for miR-200b/200c/429 and AACACU for miR-200a*/141 [84]. Recently, several other groups have reported a role for the miR-200 family in the Epithelial-Mesenchymal-transition (EMT) and in cancer cell migration, the latter by directly targeting the transcription factors ZEB1 and ZEB2, which regulate E-Cadherin, a mediator of cell-cell adhesion [84,85]. Another study [86] identified a regulatory loop between these microRNAs and ZEB transcription factors as well as the EMT inducer TGFβ. In epithelial cells, miR-200 family microRNAs and E-cadherin maintain higher level expression by repressing ZEB1, ZEB2 and TGFβ; on the other hand, in mesenchymal cells and tumors, the up-regulation of ZEB factors is triggered by TGFβ and suppresses the transcription of miR-141/200c by binding to their putative common promoter region. In our primary dataset, ZEB1 and ZEB2 were both up-regulated in six out of our eight ccRCC samples and, their expression levels were highly anti-correlated with the miR-200 family in both tumor and normal samples. As confirmation of these results, down-regulation of miR-141 and miR-200c and their function on ZEB2 in ccRCC has recently been reported [87]. We also noted that in our data, the anti-correlation between VEGFA and the miR-200 family was strongest in normal kidney tissue, suggesting that loss of this regulation may be an important factor providing a permissive environment for HIF transcriptional signaling. Our hypothesis (prediction) from these various observations is that in normal kidney, the expression level of HIF2α and its downstream targets (VEGFA, TGFβ etc) are regulated by miR-141, 200a*, 200b and 200c and the loss of this microRNA regulation, in concert with VHL loss, is responsible for activation of the HIF pathway.

**Figure 6:**
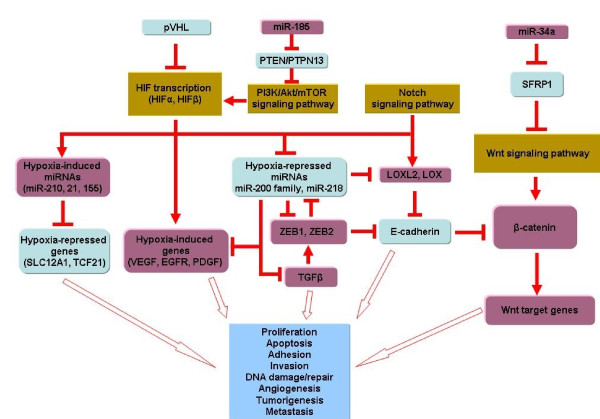
**Model of dysregulated pathways in ccRCC based on predicted microRNA/mRNA interactions and known signaling pathways from the literature**. This figure shows some of the biological pathways and regulatory interactions in normal kidney that are altered/dysregulated in ccRCC by changes in microRNA/mRNA levels. Light blue and violet/red indicates genes that are down-regulated and up-regulated in tumors, respectively, while dark golden colors show pathways.

One intriguing association which we have identified (miR-141 regulation of SEMA6A) is highly significant for therapy in ccRCC. This is because the soluble extracellular domain of SEMA6A has been engineered to effectively inhibit VEGF-mediated tumor formation [88]. Hence, our results imply that miR-141 may have a role in gene therapy. A model which summarizes our observations and integrates it with mechanisms for ccRCC dysregulation from the literature is shown in Figure 6. This model integrates our measured microRNA regulatory mechanisms with known transcriptional activity resulting from *VHL *loss and activation of the hypoxia response pathway. The pathways shown in Figure 6 are likely highly interconnecting, and this model, and the individual functional interactions it suggests, need to be validated (and probably significantly extended) by direct experimental targeting of microRNA levels and measurements of each of the predicted target genes in a larger cohort (such as is planned for The Cancer Genome Atlas (TCGA) in its next phase).

## Conclusions

The main innovation in this paper is the use of an anti-correlation analysis of microRNA/mRNA levels in two cell types from the same patient (tumor/normal cells in our case) to identify functional mRNA targets of altered microRNA. The method can obviously extend to any tissue type and might be useful in other contexts: for instance, by using microdissection to harvest cells from different cellular compartments from the same breast cancer patient, it might reveal the microRNA/mRNA regulation program which causes progression of normal epithelium to hyperplasia to DCIS to invasive disease. The method might also be useful in non-cancer studies, such as in identifying the role of microRNA and their target genes in the transition from stem cells to differentiated cells or in embryogenesis. In summary, in this paper, we have demonstrated a simple method to identify tissue specific mRNA targets of microRNA, which is expandable to many study types.

## Methods

### Primary Dataset: Tissue specimens, RNA extraction and cDNA synthesis

Eight ccRCC tissue specimens and adjacent normal kidney (NK) tissue were collected from patients at Boston Medical Center and Cleveland Clinic immediately after radical nephrectomy, processed and stored at -80°C until RNA extraction. Total RNA was extracted by homogenizing 40 milligrams of frozen tissue, followed by RNA isolation. The concentration of the purified RNA was assessed and RNA was reverse transcribed into first-strand cDNA for real-time PCR.

### Identifying the microRNA panel

Using this primary cohort of eight normal/tumor tissue pairs, microRNA expression profiling was performed in triplicate for each normal and ccRCC sample using quantitative real-time PCR in a 384-well format (System Biosciences (SBI), Mountain View, CA, USA). Expression levels were quantified using the comparative Ct (cycle threshold) method and normalized to a "housekeeping" microRNA, identified as the one whose level was most unchanged across normal and tumor samples. To identify microRNA signatures that distinguish tumor from normal tissue, we used the signal-to-noise ratio statistic [89] and computed the associated *p*-value using 1000 permutation tests with multiple hypothesis correction using false discovery rate (FDR) and *q*-values [90].

### Identifying differentially expressed mRNA

mRNA expression levels for the primary paired tumor/normal specimens were measured by hybridizing extracted RNA to Affymetrix HG-U133 Plus 2.0 arrays, and the expression data was exported by MAS5.0 and log_2 _transformed. To identify genes which strongly differentiate between ccRCC and normal kidney tissue, we use a two-fold expression cutoff and assessed their significance using permutation tests to measure *p*-values and False Discovery Rates (FDR) using SAM http://www-stat.stanford.edu/~tibs/SAM/.

### Validation on patient matched ccRCC and normal kidney

The validation cohort of 17 ccRCC tumor and adjacent normal kidney tissue samples was collected from the University of North Carolina Tumor Bank. These samples were snap frozen in liquid nitrogen, quality assured by histologic analysis of adjacent fixed sections, and stored at -80. Total RNA was extracted using the Qiagen miRNeasy Mini Kit, quantified by Nanodrop (ThermoScientific), and quality checked on an ABI Bioanalyzer (ABI). MicroRNA analysis on the validation set of 17 ccRCC tumor and surrounding NK was performed using a highly distinct platform from the experimental set: Samples were end-labeled and hybridized to a commercial densely tiled probe Agilent 8 × 15 K microRNA array. We included more samples in the validation set than in the primary set because of the lower signal/noise ratio in microRNA expression values using the Agilent chip compared to RT-PCR. The validation set of mRNA samples were co-hybridized with a commercial RNA reference (Stratagene) supplemented with a routine set of tumor genes [83] to provide a standard reference for relative expression. Hybridization was done on a commercial Agilent human 4 × 44 K cDNA array to measure expression levels.

The samples collected at Boston University and Cleveland Clinic were obtained from patients immediately after radical nephrectomy under IRB approved informed consent from all patients. The IRB approving bodies were the Boston University Medical Center Institutional Review Board and the Cleveland Clinic Regional Institutional Review Board respectively. The samples collected at the University of North Carolina Medical School were collected as a tumor banking protocol entitled Procurement of Solid Tumor Tissue (LCCC 9001) approved by the Biomedical Investigational Review Board (IRB) at the University of North Carolina Medical School.

### Validation set ccRCC mRNA measurements using quantitative RT-PCR

Total RNA from a subset of 12 of the 17 validation tumors and matched normal kidney sample pairs was also analyzed for mRNA expression of the predicted target genes VEGFA, ERBB4, SFRP1, and SLC12A1. Briefly, cDNA was prepared from 500 ng of RNA using SuperScript II polymerase, using manufacturer recommended standard buffer and temperature conditions. cDNA was analyzed by quantitative RT-PCR using commercial FAM-labeled probe sets (ABI) using standard cycle conditions. For each set of cDNAs analyzed, control 18S ribosomal subunit and beta-actin cDNA were measured for internal normal controls. Cycle threshold values were corrected by normalization to actin.

### In vitro validation of SEMA6A as a target of miR-141

Human renal cell adenocarcinoma cells (ATCC, CRL-1611) were transfected with either the negative control pre-miR or hsa-miR-141 (Ambion) using either FuGENE HD (Roche) or HyFect (Denville scientific) transfection reagents. RNA was isolated after 48 hours using the Trizol reagent (Invitrogen) and treated with DNase. cDNA was synthesized using the SuperScript III First-Strand synthesis system for RT-PCR (Invitrogen) using oligo(dT) primers according to the manufacturer's instructions. PCR was performed using GeneAmp Fast PCR master mix (Applied Biosystems) using SEMA6A primers (Sigma) for 35 cycles. The specific primers used were: SEMA6A forward: 5'-CCTGGACACCAGTTCCTGAT

SEMA6A reverse: 5'-GCAATTTTGGTAAGGCGGTA. The samples were analyzed on 1.5% agarose gel.

## Authors' contributions

The ideas for the study were developed and implemented in the labs of GB and WKR by HL, ARB and AR. The manuscript was written by HL, ARB, AR, WKR and GB. AJL provided guidance and background on the biology. The transfection assay was done in the lab of SG by YM and JO. The primary dataset was generated by DJ in the lab of LL. The validation data and lab analysis was done by ARB in the lab of WKR with significant assistance from AA. The bioinformatic analyses were carried out mainly by HL with significant help of ARB, AR, GA and MS. The required software was developed by MS. All authors have read and approved the final manuscript.

## Supplementary Material

Additional file 1 **Targets of upregulated microRNA: **This file contains the list of mRNA targets of upregulated microRNAs in ccRCC compared to normal kidney as identified by our method. Click here for file

Additional file 2 **Targets of downregulated microRNA: **This file contains the list of mRNA targets of downregulated microRNAs in ccRCC compared to normal kidney as identified by our method. Click here for file
